# High-responsivity vertical-illumination Si/Ge uni-traveling-carrier photodiodes based on silicon-on-insulator substrate

**DOI:** 10.1038/srep27743

**Published:** 2016-06-09

**Authors:** Chong Li, ChunLai Xue, Zhi Liu, Hui Cong, Buwen Cheng, Zonghai Hu, Xia Guo, Wuming Liu

**Affiliations:** 1 Institute of Electronic Information and Control Engineering, Beijing University of Technology, Beijing 100124, China; 2 State Key Laboratory on Integrated Optoelectronics, Institute of Semiconductors, Chinese Academy of Sciences, Beijing 100083, China; 3 Minzu University of China, Beijing 100081, China; 4 Beijing National Laboratory for Condensed Matter Physics, Institute of Physics, Chinese Academy of Sciences, Beijing 100190, China

## Abstract

Si/Ge uni-traveling carrier photodiodes exhibit higher output current when space-charge effect is overcome and the thermal effects is suppressed. High current is beneficial for increasing the dynamic range of various microwave photonic systems and simplifying high-bit-rate digital receivers in many applications. From the point of view of packaging, detectors with vertical-illumination configuration can be easily handled by pick-and-place tools and are a popular choice for making photo-receiver modules. However, vertical-illumination Si/Ge uni-traveling carrier (UTC) devices suffer from inter-constraint between high speed and high responsivity. Here, we report a high responsivity vertical-illumination Si/Ge UTC photodiode based on a silicon-on-insulator substrate. When the transmission of the monolayer anti-reflection coating was maximum, the maximum absorption efficiency of the devices was 1.45 times greater than the silicon substrate owing to constructive interference. The Si/Ge UTC photodiode had a dominant responsivity at 1550 nm of 0.18 A/W, a 50% improvement even with a 25% thinner Ge absorption layer.

High-current photodiodes, which receive communication signals in the near-infrared range, are highly beneficial for increasing the dynamic range of various photonic systems^1^,^2^ and simplifying high-bit-rate digital receivers^3^. The output radio-frequency signal level from these photodiodes can be increased with the response photocurrent. High-current photodiodes are particularly important components in optically-steered phased array antenna systems, because they can help the antenna to reduce its phase- and amplitude-matched electronic gain^4^,^5^,^6^. However, the conventional pin structure has a limitation in current density during high frequency operation, owing to the space-charge effect^7^,^8^.

The uni-traveling carrier (UTC) structure was designed to overcome the space-charge effect using a p-type doped absorption layer instead of a conventional intrinsic layer^9^,^10^,^11^,^12^,^13^,^14^. However, the photo-generated electrons diffuse from the p-type layer to the collection layer, resulting in a greatly reduce transit frequency. The reported transit frequency of typical UTC devices (with a 40 μm-diameter mesa and a 0.6 μm carrier diffusion length) approaches the RC frequency^9^,^15^. There are two general methods to increase the bandwidth. One is reducin the size of the device^16^,^17^,^18^,^19^. Unfortunately, smaller area of devices induces high thermal heating inside, which generates a negative feedback of the output power of these devices^10^,^20^. Monoatomic crystals such as Ge and Si have higher thermal conductivity than InGaAs and InP alloy materials^21^. Additionally, Si/Ge devices have great advantages in their compatibility with complementary metal-oxide-semiconductor (CMOS) technology and large-scale monolithic integration circuits, low cost, and low power consumption^22^,^23^,^24^,^25^,^26^. Therefore, Si/Ge uni-traveling carrier photodiodes have dramatic practical potential for high-current output applications^19^,^27^. The other method is shortening the diffusion length^28^. However, the absorption efficiency decreases with the diffusion length, for the devices with the most commonly-used vertical-illumination configuration. Vertical-illumination configuration has great advantage on easily handling by pick-and-place tools and is a most popular choice for making photo-receiver modules^29^,^30^,^31^. To our knowledge, the first vertical-illumination Si/Ge UTC device was reported by M. Piels, who demonstrated a low thermal impedance of 520 K/W and a 1 dB saturation photocurrent of 20 mA. The responsivity of their device at 1550 nm was 0.12 A/W with a 0.8-μm-thick Ge absorption layer on silicon substrate^27^. Our group reported a 40 μm-diameter vertical-illumination Si/Ge UTC device with a high responsivity of 0.18 A/W but low bandwidth of 2.7 GHz 32. Increasing the thickness of the Ge absorption layer could improve the responsivity^31^, but decrease the electron transit frequency and the response speed^34^. Therefore, it is a challenge to obtain both high responsivity and high speed at the same time in vertical-illumination Si/Ge UTC detectors.

Silicon-on-insulator (SOI) substrates have great advantages on the responsivity and bandwidth performance of Si/Ge photodiodes. First, the large difference in refractive index between the buried oxide layer (BOX) and the Si is beneficial in recycling transmission light back to the absorption layer, which is equivalent to extending the absorption length. This allows the absorption efficiency of the photodiodes to be increased without sacrificing the response speed. Second, high quality Ge film with low threading dislocation density (TDD) can be obtained on the SOI substrate by elastic deformation of the top silicon membrane, and adapt to the lattice of the Ge hetero-epitaxial file, which could increase the carrier lifetime and decrease the non-radiative recombination rate, increasing the collection efficiency^33^,^34^,^35^,^36^. Therefore, the carrier collection efficiency of the device may be enhanced using a SOI substrate. Third, the use of a SOI substrate could reduce the parasitic capacitance of the device, which would result in a better frequency response performance^37^,^38^ and a decrease in power loss^39^.

Here, we report a high-speed, high responsivity vertical-illumination Si/Ge UTC-PD based on a silicon-on-insulator (SOI) substrate. The silicon-on-insulator substrate was used to reflect transmission light for high absorption efficiency, and to improve the lattice quality of the Ge epitaxial layer to increase the efficiency of photon-generated carrier collection. The absorption efficiency of the Ge-on-SOI UTC photodiode was found to vary periodically with the thicknesses of both the BOX and Si layers, owing to the interference between the incident light and the light reflected by the BOX layer of the SOI. Moreover, the maximum absorption efficiency of the devices on SOI was found to be 1.45 times greater than that of the silicon substrate and 2.3 times greater than the minimum absorption efficiency, with the maximum light transmission of the monolayer anti-reflection coating. We achieved a 3-dB bandwidth much larger than that in our previous work^33^ by using a smaller device size of 15-μm-diameter. Meamwhile the thermal effect usually more serious in smaller devices was suppresed by using a large second meas electrde and the 1-dB compression current was 16.2 mA at 3 GH, which is similar to the 40-μm-diameter device at 1 GH^33^.

## Results

### Structure and electric field

**Figure 1:**
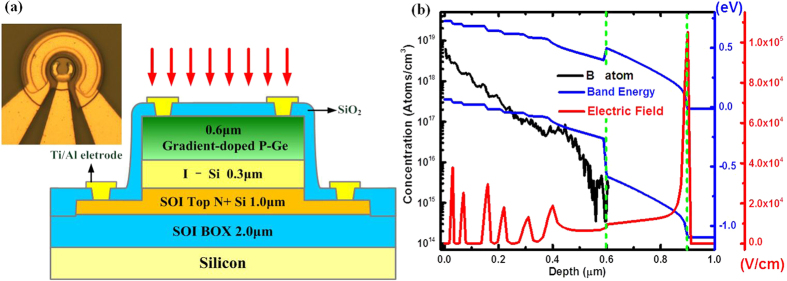
(**a**) Cross-sectional schematic view of the reported Ge-on-SOI UTC photodetector and top view of a double-mesa structure of the Ge-on-SOI UTC photodetector. The substrate was SOI with a 1.0-μm-thick n-doped top Si film and a 2-μm-thick BOX layer, the collect layer was a 0.3-μm-thick intrinsic epitaxial silicon layer, and the absorption layer was a 0.6-μm-thick epitaxial germanium layer with step gradient doping of B atoms. (**b**) The left black coordinate and curve show the doping concentration of B atoms in the Ge absorption layer step, as determined by SIMS. The etch step was nearly 0.05 μm wide, resulting in six high local electric fields to accelerate the photon-generated electrons and shorten the transmit time. In the simulation, the doping concentration in absorption layer decreased form 5 × 10^19^/cm^3^ to 2 × 10^17^/cm^3^, which was modified according to the doping parameters of SIMS measurements. The right red and blue coordinates show the electric field and band energy of our devices without bias, respectively. The peak value and width of the six local electric fields were determined by the doping concentration around the step interfaces. According to our simulation, six step gradients in the absorption layer enabled a maximum potential difference across the layer.

Expermentally, the open circle shaped top mesa electrode was desighed to improve the success rate of lift-off. And the large second mesa electrode with a 10-μm-width as shown in the inset of Fig. 1(a) was used to supress the thermal effect to ensure a high compress current. Figure 1(a) shows a cross-sectional schematic view of a Si/Ge UTC photodiode based on a commercially available SOI substrate with 1.0-μm-thick n-doped Si and 2-μm-thick BOX layers. A step gradient doped profile was employed, which enabled the generation of several regions with high local electric field to further decrease the transit time of the photo-generated electrons, as the red curve shown in Fig. 1(b). A simulated band-gap diagram and electric field distribution at 0 V of such a device are illustrated in Fig. 1(b) by the blue and red curves, respectively, calculated after modifying the doped parameters according to the results of secondary ion mass spectrometry (SIMS) measurements. The built-in electric field was generated from the differences in the doped concentration. Each abrupt change in doped concentration corresponded to an electric field peak. The photon-generated electrons were accelerated in the Ge absorption layer and gained kinetic energy to pass through the Si/Ge heterojunction barrier under the action of the built-in electric field. A larger electric field and thus lower transit time can be obtained as compared to conventional linear-gradient-doped absorption layer^11^,^40^,^41^.

### Responsivity characterization

The responsivity of a vertical-illumination photodiode is limited mainly by a combination of three factors: (1) the coupling efficiency determined by the top anti-reflection coating, (2) the collection efficiency of the photon-generated carriers^42^, (3) the absorption efficiency of the Ge layer. Generally, only the light coupled into the absorber (*P*_*0*_) can be converted into electron-hole pairs. To maximize the coupled light and minimize the antireflection loss, the thickness of the top anti-reflection coating should be N·(λ/4n), due to destructive coherence inside the coating. In our device, N = 3, n is the refractive index of the coating (SiO_2_: ~1.47)^43^ and the transmission is about 0.94. The carrier collection efficiency of Si/Ge UTC photodiodes is mainly determined by the design of the electric field inside the devices and by the recombination caused by defects inside the Ge layer and at the hetero-interface^44^,^45^. Absorption efficiency is generally dependent on the absorption coefficient and absorption length. The absorption coefficient of Ge at 1550 nm could be significantly affected by the tensile-strain, and generally changed from 840 /cm (0% tensile-strain) to 4570 /cm (0.25% tensile-strain)^46^.

Assuming a 100% carrier collection efficiency, the absorption length is generally equal to the thickness of the absorber. Therefore, the power inside the absorption layer *P*_*ab*_ can be expressed by *P*_*ab*_* = P*_*0*_(*1*−*e*^−*aD*^), where *α* is the absorption coefficient of the absorber and *D* is thickness of the absorber. However, the introduction of the SOI substrate was expected to cause recycling of the transmission light and improve the light absorption length and efficiency by the reflection of the substrate transmission light. The new absorption power is:
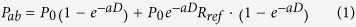
where *R*_*ref*_ is the reflection coefficient of the SOI substrate, and *R*_*ref*_ is the reflection coefficient of the top coating film and Ge film.

**Figure 2:**
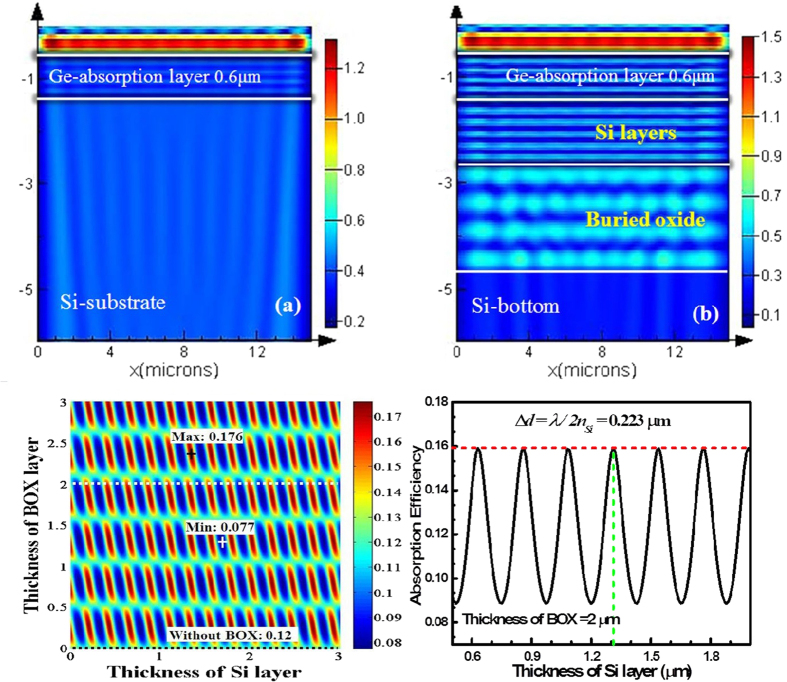
The wavelength of the incident light was 1550 nm, and the absorption efficiency of the Ge material was 1000/cm. Optical power distribution inside Si/Ge UTC devices grown on (**a**) Si substrate and (**b**) SOI substrate with the same imput light, same top anti-reflection coating and same Ge layer. The only difference between the two devices was the inserted 2-μm-thick BOX layer, and the thickness of the silicon collection and contact layers was 1.3 μm. The scale bar illustrates the optical power. Because of the coherence effect between the incident light and reflected light of the BOX layer, the optical power inside the Ge, Si, and BOX exhibited a periodic enhancement distribution. The period was determined by the refractive index and the wavelength of the incident light. The light inside the Si bottom layer of the SOI substrate and inside silicon substrate was the unemployed light of the devices. Obviously, that in the SOI was much lower than that in the silicon substrate. Thus, the SOI substrate was more beneficial for higher light absorption by Ge, compared with the Si substrate. When the transmission of the anti-reflection coating was maximum, (**c**) Relationship between the absorption efficiency of Ge-on-SOI UTC photodiode and the thicknesses of the BOX and silicon layers. Using these thicknesses of the BOX and silicon as ordinate and abscissa, respectively, the absorption efficiency is mapped in colored points in a two-dimensional coordinate plane. The bottom black dashed line represents the absorption efficiency of the devices without BOX layer, which was 0.12. The other two special points are the maximum of 0.176 with a 1.11-μm-thick Si layer and a 1.325-μm-thick BOX, and the minimum of 0.077 with a 2.13-μm-thick Si layer and a 1.84-μm-thick BOX. (**d**) Relationship between the absorption efficiency and the thickness of the silicon layer, when the thickness of the BOX is the general commercial value of 2 μm. The absorption efficiency changes periodically with the thickness of Si layer because of constructive interference. The period is *T = λ/2n,* which is 0.223 μm for the silicon layer, and the maximum efficiency is 0.159 when the silicon thickness is 1.31 μm, which is close to the structural parameter of our device.

A commercial finite-difference-time-domain (FDTD) simulation package was used to compare the detailed optical power distributions in the Si/Ge UTC photodiodes on the Si and SOI substrates with the same incident light, top anti-reflection coating and Ge layer, as shown in Fig. 2(a,b). The scale bar is for the optical power. The optical power inside the Si bottom layer of the SOI substrate is obviously much lower than that in the silicon substrate. Therefore, the SOI substrate is more beneficial for higher light absorption by the Ge layer than the Si substrate.

The relationship between the absorption efficiency and the thickness of the BOX and silicon layer was analysed and calculated by the scattering matrix methods using Matrix Laboratory (MATLAB). When the transmission of the anti-reflection coating was maximum, the absorption efficiency varied periodically with the thickness of the substrates, as shown in Fig. 2 (c). The SOI substrate is a compliant substrate, which could further release the built-in strain^33^,^52^,^47^. Therefore, it is supposed that the theoretical absorption coefficient at 1550 nm of our epitaxial Ge is 1000/cm with a lower than 0.13% tensile-strain, which is the tensile-strain of the Ge epitaxial layer on silicon substrate in our laboratory^48^,^49^,^50^. The absorption efficiency of the device was 0.121 with a 0.8-μm-thick coating film without BOX. The absorption efficiency increased to 0.176 with a 1.11-μm-thick Si layer and a 1.325-μm-thick BOX. However, the general thickness of the BOX layer in commercial SOI substrates is 2 μm, and the absorption efficiency varied periodically with the thicknesses of Si shown in Fig. 2(d). Because of the constructive interference between the reflected and input waves, the thickness period (*T*) is *T = λ/2n,* where *λ* is the input wavelength and *n is* the refractive index of the transmission media. In our device, the silicon thickness cycle was 0.223 μm with a Si thickness of 1.3 μm. Thus, the theoretical maximal absorption efficiency is about 0.157, and the ideal responsivity is 0.196 A/W with 100% photon-generated carrier collection efficiency and a 1000 /cm absorption coefficient at 1550 nm.

**Figure 3:**
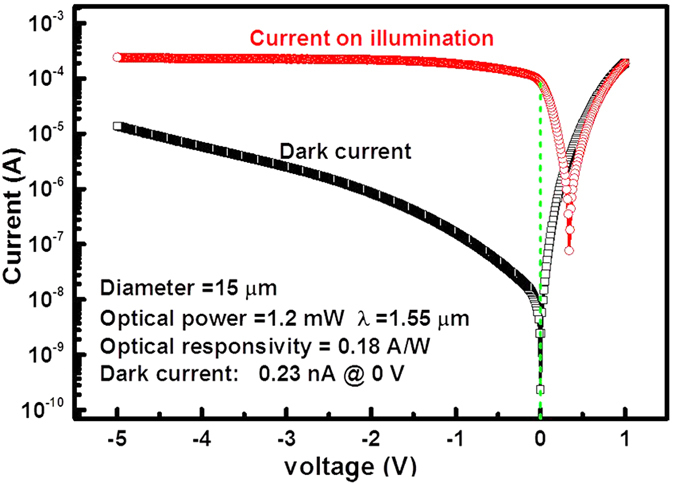
Current-voltage characteristics of the 15-μm-diameter device without illumination and under laser irradiation with an input optical power of 1.2 mW at 1550 nm. The dark current without bias was 0.23 nA. When the reverse bias was increased to 1 V, the dark current rose to 58 nA, corresponding with a current density of 96.3 mA/cm. The optical responsivity was 0.18 A/W at 1550 nm. The dashed line indicates that there was a saturation of the optical responsivity values at 0 V bias, which indicates that this photodetector configuration allowed nearly complete photo-generated carrier collection without bias.

The experimentally determined device current for the 15-μm-diameter photodiodes, without illumination and with the normally incident light on the top surface, are shown in Fig. 3. The dark current was 58 nA under a reverse bias of 1 V, which corresponds to a current density of 96.3 mA/cm^2^. The minimum dark current density was approximately 61.9 mA/cm^2^ of the 40-μm-diameter device at −1 V. The dark current could be further reduced by appropriate thermal processing to decrease the threading dislocation density around the Si/Ge interface^51^,^52^,passivation, or application of a guard-ring around the sidewall.

**Figure 4:**
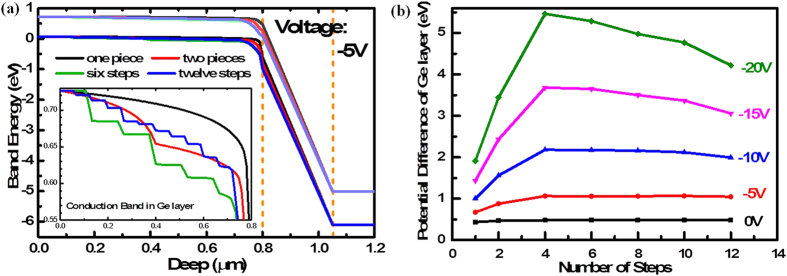
(**a**) The band energy of the Si/Ge UTC photodiodes with a linear-gradient-doped (one piece), linear-gradient-doped with a step (two pieces), 6-step-and 12-step- gradient-doped Ge absorption layer at -5 V bias. the inset figure show the conduction band in Ge layer. The potential difference of the linear gradient doped Ge layer is lower than the step-gradient-doped structure. (**b**) The potential difference of Ge layer with one step, 2-step-, 4-step-, 6-step-, 8-step-, 10-step-, and 12-step-gradient-doped at different biases. The peak potential difference is 0.48 eV at 0 V with 4-step-gradient-doped, approximate 8% of the total potential difference of the devices. However, when the bias voltage increased to −20 V, the potential difference of Ge layer is 5.46 eV, almost 21% of the device potential difference. Actually, limited by the technology of the *in-situ* doped epitaxial, the Ge layer of our device is six steps gradient doped.

At a reverse bias of 1 V, the optical responsivity was 0.18 A/W at 1550 nm with a 0.6-μm-thick Ge layer, 50% higher than in the previously reported Si/Ge UTC devices (*R* = 0.12 A/W with a 0.8-μm-thick Ge layer)^27^. In Fig. 4, The photocurrent is flat over a wide range of reverse bias voltage, and the optical currents around zero bias voltage show that a built-in electric field is already established within the p-type Ge layer and Si layer without applying a bia^53^. Because of the carrier recombination, this measured responsivity is little lower than the theoretical results.

### 3-dB bandwidth characterization

The bandwidth of common photodiode is limited mainly by the resistor–capacitor (RC) bandwidth (*f*_*RC*_) and the carrier transit-time-limited bandwidth (*f*_*t*_) in the active region^54^. Particularly, for UTC devices, the transfer time of electrons in the p-type absorption layer is more important than the RC time constant, owing to their low diffusion velocity^16^. Based on the principle of conservation of energy, the electrical potential difference across the absorption layer determines the change in kinetic energy of the carriers. A high kinetic energy in the absorber could shorten the transfer time of the electrons. Based on the direct relationship between the doping difference across the doped junction and the electrical potential difference, a step gradient-doped region was introduced in the absorption layer to increase the potential difference across the Ge layer. Figure 4(a) shows the band energy of the devices with a linear-gradient-doped, linear-gradient-doped with 1-step, 6-step-and 12-step- gradient-doped absorption layer. The doping profile is uniform within each step region. The electrical potential difference in the step-gradient doped layer is higher than that in the conventional linear gradient doped layer. We also calculated the potential differences of Ge layer with one step, 2-step-, 4-step-, 6-step-, 8-step-, 10-step-, and 12-step- gradient-doped at different bias, shown in Fig. 4(b). The maximal value of the potential difference value can be achieved with a 4-step-gradiant-doped Ge layer. However, limited by the technology of the *in-situ* doped epitaxy, the Ge layer of our device is six steps gradient doped. Therefore, we can assume that the photo-generated electrons drifted with saturation velocity (*v*_*s*_) in the p-type absorption layer of our devices owing to the high potential drop across the absorber, and then cross the collection layer with thermionic emission velocity (*v*_*th*_), because of the effect of the velocity overshoot^9^. Then, the carrier transit frequency can be approximated by the following equation:
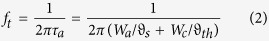
where *v*_*th*_ is the thermionic emission velocity, and *v*_*s*_ is the saturation velocity.

**Figure 5:**
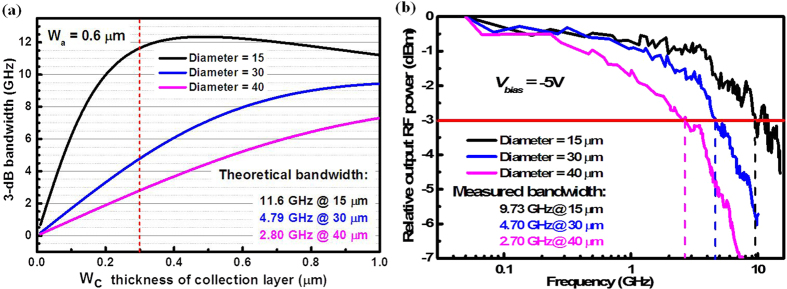
Frequency responses for UTC photodiodes with diameters of 15, 30, and 40 μm under 1550 nm incident light: **(a**) theoretically predicted values for a series resistance of 100 Ω, which was measured using the slope of the I-V curve at the positive bias of 0.26 V and on the assumption that the photo-generated electrons traveled across the p-type absorption layer by drifting with saturation velocity (*v*_*s*_), and across the collect layer by drifting with thermionic emission velocity (*v*_*th*_). (**b**) The 3-dB bandwidth was measured with a vector network analyser with a bias of −5 V. The theoretical *f*_*3*_* *_*dB*_ values are almost consistent with the experimental results except for the 15-μm-diameter device, whose difference may have resulted from the higher series resistance of smaller size devices caused by the complicated fabrication processes.

Moreover, there are no depletion or depletion-layer capacitance in absorption layer due to same material, same bandgap and same doping type around the step-gradient doped interfaces. The parasitic capacitance, resulting from leak current and threading dislocation in the Ge epitaxial layer, is small enough to be ignored compared with the junction capacitance. Therefore, and *f*_*RC*_ can be approximated using:
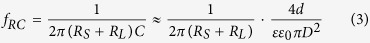
where *W*_*c*_ is the collection layer thickness, *W*_*a*_ is the absorption layer thickness, *d* is the thickness of depletion region, *D* is the mesa diameter, *R*_*L*_ is the load resistance (50 Ω in this case), *R*_*S*_ is the series resistance, and *ε* and *ε*_*0*_ are the relative and vacuum permittivity, respectively. Series resistance arises from the resistance of the contacts and the resistance of the un-depleted region, which could be inferred by the forward I-V characteristic curves. The series resistance is inversely proportional to the area of the device, approximately equal to the slope of the I-V curve at positive bias^55^. Here, the used series resistance of the device is about 100 Ω, which was the measured resistance of the 40 μm-diameter device^33^. The theoretical values of *f*_*3*_* *_*dB*_ with various diameters are shown in Fig. 5(a). The consistency between the experimental and theoretical results showed that the step gradient-doped design was able to make the carriers drift in the absorber with a saturation velocity and efficiently increase the transit frequency of the UTC photodiodes. Because the series resistance is inversely proportional to the area of the device, the actual bandwidth of the 15-μm-diameter device is little lower than the calculated value, as shown in Fig. 5(b). The bandwidth of our photodiodes was mainly limited by the high contact resistance of n-type Si layer, which were measured to be .1.75 × 10^−3 ^Ω·cm^2^, and 2~3 orders of magnitude more than the normal contact resistance^56^. Specific contact resistance is a function of the barrier height, doping concentration and temperature for a fixed semiconductor material. Therefore, some approaches, such as Al contact metal instead of Ti/Al, ions re-implantation in metal contact region and wet etching before metallizing to get rid of the etching residue or oxide film on the semiconductor, could significantly reduce the series resistance of our photodiode. Theoretically, the 3-dB bandwidth of our 15 μm-diameter could be as high as 51 GHz with a 1 Ω series resistance about 30 GHz higher bandwidth than previously reported bandwidth^33^. More work to improve this bandwidth is underway. Additional, the parasitic capacitance and series resistance of the SOI substrate is lower than silicon substrate. Therefore, the bandwidth of devices based on SOI should be higher than based on silicon substrate, theoretically.

### Saturation characterization

**Figure 6:**
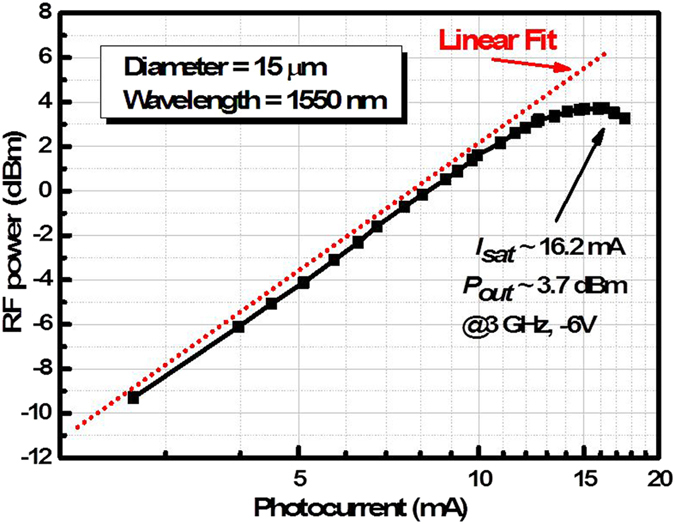
Results of large signal −1-dB compression photocurrent measurement for the 15-μm-diameter Si/Ge UTC photodiodes. The incident light had a wavelength of 1550 nm, 100% modulation depth, and modulation frequency fixed at 3 GHz. The saturation current was 16.2 mA at a reverse bias of 6 V, and the output RF power was 3.7 dBmW. The saturation current and RF power were further increased with the reverse bias.

The device saturation current was obtained using large signal measurements, as shown in Fig. 6. A 100% modulation depth tone was fixed at 3 GHz. The 15-μm-diameter device exbibited a 3 dB bandwidth of 9.7 GHz. The 1-dB compression currents was 16.2 mA at reverse bias voltages of -6 V. The fixed modulation frequency of the 40-μm-diameter device in ref. 33 was 1 GHz. The a 3 dB bandwidth was 2.7 GHz, and the saturation current was also 16.2 mA under −7 V bias. Three main effects limit the photocurrent saturation of the device: voltage drop and swing, thermal effect and space-charge effect^15^. The high modulation frequency could increase the voltage drop and the small area of device could enhance space-charge effect, which both decrease the compression current of the photodiode. However, the large second mesa electrode of the 15-μm-diameter device had great benefits on the thermal dissipation and low series resistance. Therefore, our 15-μm-diameter device have a similar -1-dB compression current yet with a higher modulation frequency, higher bandwidth and a lower working voltage, compared with the 40-μm-diameter device in ref. 33. Moreover, The saturation of the Ge-on-SOI UTC photodiodes could be further improved by suppressing the thermal effects^56^,^57^ by substrate thinning^27^ and decreasing the thickness of the BOX layer.

## Discussion

The demonstrated on-chip performance of the present high-responsivity vertical-illumination Si/Ge uni-traveling-carrier photodiodes paves the way for all kinds of vertical-illumination Si/Ge photodetectors with high responsivity, and high-quality epitaxial germanium. It will also allow the realization of large-scale monolithic integrated microwave optoelectronic antenna systems with low cost and low power consumption. The use of silicon-on-insulator substrate for Si/Ge UTC photodiodes offers advantages in reflecting the light to increase the absorption efficiency of the input optical signal, and improving the lattice quality of Ge epitaxial layer to increase the efficiency of photon generated carrier collection. Because of the constructive interference between the incident light and the light reflected by the buried oxide layer of the SOI, the maximum absorption efficiency of the devices on SOI substrate was 1.45 times greater than that obtained with silicon substrate and 2.3 times greater than the minimum absorption efficiency with a maximum transmission of the anti-reflection coating. The photodiodes showed a responsivity of 0.18 A/W at a wavelength of 1.55 μm, which is 50% higher than the previously reported Si/Ge UTC devices using Si substrates. Furthermore, use of a step gradient-doped absorber caused the carriers to drift with a saturation velocity in the absorber, which efficiently increased the transit frequency of the UTC photodiodes. As a result, the 3-dB bandwidth of the 15-μm-diameter device was improved to 9.72 GHz under a −5 V bias voltage. The 1-dB compression current of the device was 16.2 mA at 3 GHz. Hindered by the high series resistance and the area of the device, the performance of our device is not yet comparable to the more mature III/V devices. Nevertheless, this study introduces the great potential of Si/Ge UTC photodiodes to high-speed microwave photonic monolithic integrated applications.

## Methods

### Silicon and germanium film growth and characteristics

After the growth of the intrinsic Si layer at 750 °C by cold-wall ultra-high vacuum chemical vapour deposition on the SOI substrate using a source gas of pure Si_2_H_6_ (UHV-CVD), a brief interruption was introduced to decrease the growth temperature to 290 °C for the growth of the 60-nm-thick p-doped Ge buffer layer. A 600-nm-thick boron-doped Ge layer was then grown on the top as the absorption layer at 600 °C using pure GeH_4_ and diluted B_2_H_6_ source gases. Six boron-doping concentration steps were made in the Ge absorption layer.

### Fabrication and characterization of photodiodes

Circular Ge layer mesas for normal incidence Si/Ge UTC photodiodes with diameters ranging from 15 to 40 μm were defined by standard photolithography and inductively coupled plasma (ICP) etching. The second mesa was etched to the 2-μm-thick buried oxide layer. The double mesa layout significantly reduced the parasitic capacitance. Top and bottom contacts were lithographically defined on evaporated Ti/Al and a rapid-thermal-annealing (RTA) process was carried out for impurity activation. A passivation/antireflection coating was deposited by plasma enhanced chemical vapour deposition (PE-CVD). Windows for the metal contacts were opened by C_4_F_8_ ICP etching. The metal pad was evaporated and lifted off. A micrograph of a photodiode with a 15-diameter top mesa is shown in Fig. 1(b). The current-voltage characteristics of our device was measured using an Agilent B1500A semiconductor parameter analyser on a probe station at room temperature. The photocurrent-voltage characteristics were obtained under laser irradiation at a wavelength of 1550 nm with power of 1.2 mW.

### Saturation measurements

The device saturation current was obtained using large signal measurements. A heterodyne technique using two free-running lasers at 1550 nm was used and a modulation index was ultimately obtained. A 100% modulation depth tone was fixed at −3 GHz for measurement of the 15-μm-diameter device.

## Additional Information

**How to cite this article**: Li, C. *et al*. High-responsivity vertical-illumination Si/Ge uni-traveling-carrier photodiodes based on silicon-on-insulator substrate. *Sci. Rep.* **6**, 27743; doi: 10.1038/srep27743 (2016).
